# Label-free assessment of a microfluidic vessel-on-chip model with visible-light optical tomography reveals structural changes in vascular networks

**DOI:** 10.1039/d5lc00927h

**Published:** 2026-01-30

**Authors:** Devin Veerman, Carlos Cuartas-Vélez, Tarek Gensheimer, Tomas van Dorp, Andries van der Meer, Nienke Bosschaart

**Affiliations:** a Applied Stem Cell Technologies, Department of Bioengineering Technologies, TechMed Centre, University of Twente Enschede The Netherlands d.veerman@utwente.nl; b BIOS Lab on a Chip Group, MESA+ Institute, Technical Medical Center, Max Planck Institute for Complex Fluid Dynamics, University of Twente Enschede The Netherlands; c Biomedical Photonic Imaging Group, Department of Bioengineering Technologies, TechMed Centre, University of Twente Enschede The Netherlands n.bosschaart@utwente.nl

## Abstract

Microvascular dysfunction is characterized by impaired structure and function of small blood vessels, contributing to disease-related tissue and organ damage, such as in the retina. Optical coherence tomography is a widely used clinical technology to detect, monitor and diagnose disorders of the retina and choroid, such as diabetic retinopathy, macular degeneration, and various inherited retinal diseases. Currently, there are limited experimental platforms that correlate observed changes in clinical metrics with underlying mechanisms of disease progression. Organ-on-chips have the potential to offer a platform for correlative studies. Previous studies have demonstrated that the three-dimensional complexity of the microvasculature can be captured in a vessel-on-chip. Yet, current vessel-on-chip imaging analysis is based on end-point read-outs that provide limited dynamic information and do not have direct correlation with imaging techniques used in the clinic. Therefore, there is a need for clinically relevant, label-free, real-time imaging technologies. In this work, we show that optical coherence tomography can fulfill this need by providing non-invasive, label-free imaging of vascular networks-on-chip. We show that optical coherence tomography can detect and can be used to quantify changes in vascular network structures over multiple days, both during vascular network development and in response to disease-associated conditions. Our results indicate that optical coherence tomography has the potential to become a standard read-out for monitoring dynamic processes in organ-on-chips. In the future, these read-outs may enable the correlation of clinical metrics, thereby providing deeper insights in the pathophysiology of diseases, for example of the retina.

## Abstract 2

Optical coherence tomography is a label-free imaging technique that can provide structural readouts in a vessel-on-chip.

## Graphical abstract

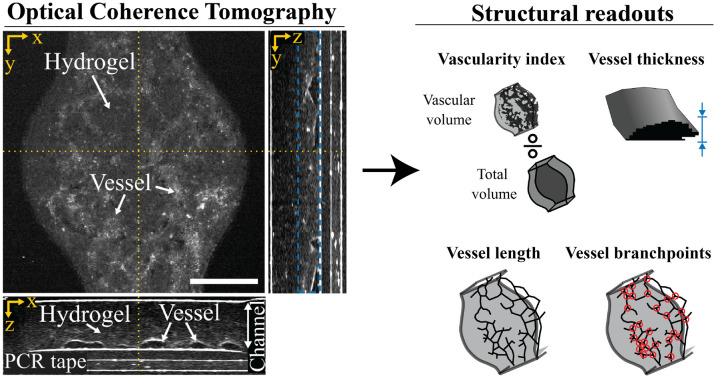

## Introduction

Microvascular dysfunction covers a broad range of conditions characterized by impaired structure and function of small blood vessels resulting in severe tissue and organ damage. Vital organs are affected by this disease, such as the heart, lung, kidney, brain, and retina. Microvascular dysfunction is hypothesized to be a chronic disease that is exacerbated with age and advances with vascular risk factors.^73^

In the case of the retina, diabetic retinopathy (DR) arises as a microvascular complication attributable to diabetes mellitus, a common vascular risk factor. In the early, non-proliferative stage of DR (NPDR), advanced glycation end products derived from elevated blood glucose trigger a cascade reaction that causes oxidative stress damage and neurodegeneration to retinal cells. Neurodegeneration also damages endothelial cells, induces apoptosis in pericytes, and blocks capillaries. Consequently, these reactions disrupt and regress vascular networks, leading to progressive vision loss.^1^ Another retinal disease in which the microvascular component is affected, is age-related macular degeneration (AMD). AMD originates from malfunction of crucial retinal processes that involve the photoreceptors, retinal pigment epithelium, Bruch's membrane, and the choroid.^2^ At any stage of the disease, AMD may evoke the formation of new blood vessels in the retina as a compensation for the effect of extracellular depositions (drusen), inducing choroidal neovascularization (CNV). This process is known as wet-AMD. During the exudative phase of wet-AMD, newly formed vessels produce accumulation of fluids and/or hemorrhage, leading to central vision loss.

Interestingly, onset and progression of retinal diseases, such as DR and AMD, can be tracked in the clinic using optical coherence tomography (OCT). OCT uses a broadband light source to produce three-dimensional (3D) images of highly scattering samples such as biological tissue.^3^ Due to its non-invasive nature, OCT is the clinical standard in assessing pathologies that manifest in the posterior segment of the eye.

For early disease detection, ophthalmologists rely on qualitative and quantitative changes of the morphological properties of the retina, choroid and sclera.^4–7^ More specifically, the choroid comprises a set of vascular networks that ensure proper blood flow to the eye. In general, retinal diseases detectable with OCT encompass several symptoms that manifest as alterations in the choroidal microvascular network, either through regression (*e.g.* NPDR) or proliferation (*e.g.* wet-AMD). Therefore, important parameters of the choroidal microvascular network change under disease conditions, such as vascularity, morphology, and thickness.^8,9^ In those cases, quantitative choroid metrics (*e.g.* choroidal vascularity index, choroidal thickness, blood vessel diameter and vascular connectivity) provide accurate assessment of choroidal health.^10–13^ However, the correlation between quantitative choroid metrics and eye diseases remains unclear due to the influence of external, confounding factors (*e.g.* age, sex, refractive error, anterior chamber depth, lens thickness).^14,15^ As a consequence, there is a need for biological platforms that allow controlled testing of quantitative and qualitative choroid metrics during the development of eye diseases. To overcome that limitation, organ-on-chip technologies, which are capable of mimicking specific organ functions have drawn attention in recent years.^16–18^

Organ-on-chip (OoC) devices are microphysiological systems in which human tissues are cultured in an integrated, controlled microenvironment.^19,20^ This property allows organs-on-a-chips to systematically emulate the underlying factors involved in disease expression, progression and treatment.^18^ In the case of the eye, multiple retina-on-a-chip models have been reported that integrate neural cells, photoreceptors, epithelium and microvasculature.^21^ Retinal disorders associated with DR and AMD have been recapitulated using inner and outer blood-retinal barrier-onchip models.^22–24^ Those models have demonstrated that disease hallmarks – vascular regression in non-proliferative DR and CNV in AMD – are reproducible on-chip. Despite progress in disease modeling, quantitative measurements of the vascular networks in these retina-on-chip models, and in OoC models in general, in real-time are still limited by the imaging tools. Consequently, current measurements are based on end-point analysis that requires chemical fixation, immunofluorescent labeling, and time-consuming confocal microscopy imaging.^25^ Additionally, since the development of a vascular network within microfluidic chips is a spontaneous and dynamic process, comparing samples at different time points is potentially biased.^26,27^ As a result, a label free, real-time imaging technology that allows quantitative analysis during network formation and cell maturation would be of great added value.

OCT has the potential to fill this gap by providing real-time measurements of OoC properties. This potential has been explored in studies incorporating OCT as a read-out. Existing studies demonstrate the use of OCT in airway-on-chip, retina-on-chip and vessel-on-chip devices.^26,28–30^ Those cases have provided quantitative properties of cilia motion and mucociliary transport, analysis of microvessel quality and diameter, temporal properties of thrombus formation, and flowmetry.^26,28–30^ However, most of these applications leveraged the analysis of structural or temporal changes of the OCT signal, while the volumetric morphological properties of the tissues were disregarded or limited by a small imaging volume. Moreover, monitoring of disease processes with OCT will also require implementation and demonstration of measurement over longer periods of time.

The purpose of the present study is to address the challenges related to imaging OoCs. Here, we explore the versatility and robustness of high-speed, live tissue culture and label-free OCT imaging in an induced pluripotent stem cell (iPSC)-derived 3D vessel-on-chip (VoC). We will demonstrate that OCT can provide high-resolution images of the vascular network and can track vessel development over time in both healthy control and cases in which vessel regression and proliferation are detectable. Moreover, we will show that these images can be used to quantify important vessel parameters in 3D, such as the vascularity index, vessel length, vessel thickness, and number of branches in a network. In addition, we will compare the vessel structures found with OCT imaging with those of confocal microscopy.

## Materials and methods

### Cell culture

#### Induced pluripotent stem cell culture

The induced pluripotent stem cell (iPSC) line IPS17-00041 was obtained from the Radboudumc Stem Cell Technology Center (SCTC, Radboudumc, Nijmegen) and maintained in Essential 8 medium (Gibco, USA) on vitronectin-coated 6-well plates (Gibco, USA). Before differentiation, induced pluripotent stem cells were washed in Dulbecco's phosphate-buffered saline (DPBS, Gibco, USA), detached from the surface using 0.5 mM ethylenediaminetetraacetic acid (EDTA, Invitrogen, USA) at 37 °C with 5% CO_2_, and reseeded in the required density on Matrigel-coated 6-well plates (Corning, USA).

#### Endothelial cell differentiation

Differentiation from pluripotent stem cells to endothelial cells (ECs) was conducted according to an optimized protocol originally described by Orlova *et al.*^31^ In short, iPSCs were seeded on Matrigel-coated 6-well plates (Corning, USA) at a density of 40 000 cells per ml in Essential 8 medium supplied with RevitaCell (Gibco, USA). The next day, mesoderm induction was initiated by adding 8 μM CHIR99021 (Axon Medchem, The Netherlands) in BPEL medium.^31^ On day 3, vascular specification was induced by adding 50 ng ml^−1^ vascular endothelial growth factor (VEGF) (Miltenyi Biotec, Germany) and 10 μM SB431542 (Tocris Bioscience, UK) in BPEL medium to the cells. On day 9, endothelial cells were isolated using Dynabeads CD31+ endothelial cell (Invitrogen, USA) and expanded until confluent in gelatin-coated (Sigma-Aldrich, USA) culture flasks with EC-SFM full medium.^31^ Generated iPSC-ECs were detached from the surface using TrypLE 1× (Gibco, USA) at 37 °C with 5% CO_2_, placed in cryovials with CryoStor CS10 (STEMCELL Technologies, Canada), and stored in liquid nitrogen until further use.

#### Vascular smooth muscle cell differentiation

Differentiation from pluripotent stem cells to vascular smooth muscle cells (VSMCs) was adapted from a protocol developed by Bulut *et al.*^32^ Briefly, iPSCs were first differentiated to neural crest cells (NCCs) by seeding at a density of 5000 cells per ml on Matrigel-coated 6-well plates in Essential 8 medium supplied with RevitaCell. After 2 days, neural crest induction was initiated by supplying 8 μM CHIR99021, 10 μM SB431542 (Tocris Bioscience, UK), and 10 ng mL^−1^ bFGF-2 (Miltenyi Biotec, Germany) to BPEL medium.^32^ The iPSC-NCCs were differentiated and expanded to P3, placed in cryovials with CryoStor CS10, and stored in liquid nitrogen. Thereafter, iPSC-NCCs were thawed and seeded on gelatin-coated 6-well plates. The next day, medium was changed to BPEL supplied with 2 ng mL^−1^ TGF-β3 (PeproTech, USA) and 10 ng mL^−1^ PDGF-BB (PeproTech, USA). iPSC-VSMCs were differentiated and expanded until day 8, detached from the surface using TrypLE 1× at 37 °C with 5% CO_2_, placed in cryovials with CryoStor CS10, and stored in liquid nitrogen.

#### Microfluidic chip fabrication

**Fig. 1:**
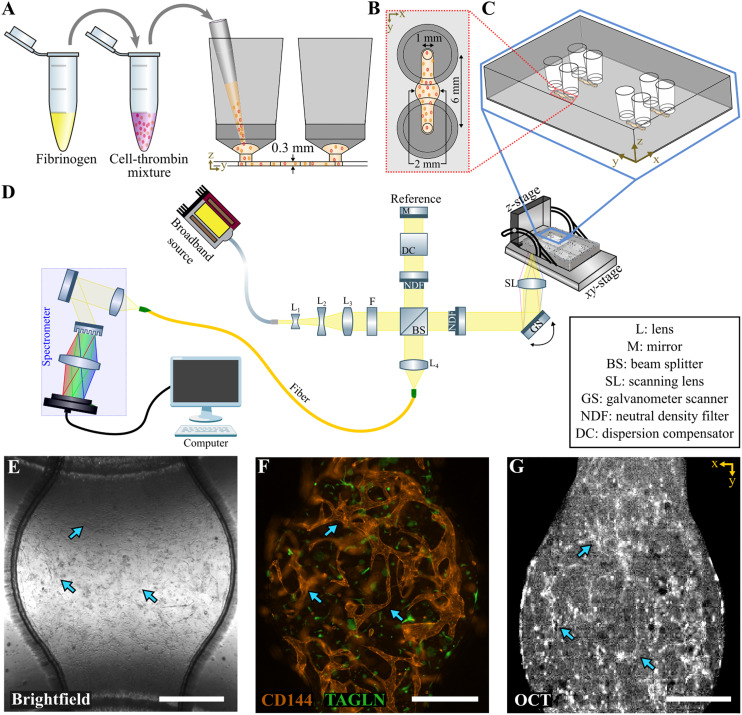
Methods for loading and visualizing vascular networks in the vessel-on-chip. A) Fibrinogen is mixed with endothelial and vascular smooth muscle cells suspended in culture medium with thrombin. This mixture is loaded in the microfluidic chip (side view). B) Top view of the chip which is 6 mm long from inlet to outlet. The inlet and outlet have a 1 mm diameter, and a 2 mm circular core in between. C) One-quarter, with each four individual addressable microfluidic chips, fit in the well-plate holder to potentially measure 16 chips simultaneously. D) Schematic view of the optical coherence tomography setup combined with the vessel-on-chip in a standardized well plate. E) Bright-field image showing vascular network on chip. F) Wide-field fluorescence image of the vascular network on chip with endothelial cells (CD144/VE-cadherin) and vascular smooth muscle cells (TAGLN/SM22). G) Raw optical coherence tomography image of the vascular network on chip. Blue arrows indicate the same location in all imaging modalities. In E–G, scale bar = 500 μm.

Microfluidic chip design was inspired by S. Zhang *et al.*^33^ Molds for the microfluidic chip were designed in SolidWorks (Dassault Systemes, France) and printed using clear v4 resin on the Form 3B+ printer (Formlabs, USA). After washing and post-curing, the molds were covered with polydimethylsiloxane (PDMS) mixed with the curing agent in a ratio of 10 : 1, respectively (Sylgard 184, Dow Inc., USA), and left to cure overnight at 65 °C (Fig. 1A–C). PDMS parts were removed from the molds and assembled by activating its surface using air plasma at 50 Watt for 60 seconds (Cute, Femto Science Inc., South Korea). The VoC bottom layer was sealed using ThermalSeal RTS adhesive tape (Excel Scientific Inc., USA). The assembled VoCs were then sterilized using air plasma at 50 watt for 90 seconds, left overnight at 65 °C to restore hydrophobicity.

#### Vascular network formation

The iPSC-VSMCs were thawed and matured until day 12 in BPEL supplied with 2 ng mL^−1^ transforming growth factor (TGF)-β3 and 10 ng mL^−1^ platelet-derived growth factor (PDGF)-BB. The iPSC-ECs were thawed and matured until confluent in EC-SFM full medium.^31^ Both cells were washed in DPBS, detached from the surface using TrypLE 1× at 37 °C with 5% CO_2_, and resuspended in endothelial growth medium 2 (EGM-2, PromoCell GmbH, Germany) to reach a concentration of 10 million cells per mL and 2 million cells per mL for the iPSC-ECs and iPSC-VSMCs, respectively. Both cell suspensions were then mixed 1 : 1 to obtain a 5 : 1 ratio of iPSC-ECs to iPSC-VSMCs, respectively.

In the meantime, fibrinogen (Sigma-Aldrich, Germany) and thrombin (Sigma-Aldrich, Germany) were thawed on ice and fibrinogen was diluted in DPBS to a working concentration of 10 mg mL^−1^. The thrombin was added to the cell suspension to obtain a thrombin concentration of 5 units per mL. Fibrinogen was then added in equal amounts to the cell–thrombin mixture to obtain a final concentration of 5 mg mL^−1^ fibrinogen and 2.5 units per mL thrombin. This mixture was then resuspended quickly, injected into the inlets of the microfluidic chip (Fig. 1A), and immediately placed in the incubator set at 37 °C with 5% CO_2_ for 25 minutes to ensure fast hydrogel crosslinking. These steps were repeated until all microfluidic chips were seeded.

After incubation, EGM-2 medium supplied with 2.5 μg mL^−1^ aprotinin (Sigma-Aldrich, Germany) and 50 ng mL^−1^ VEGF was added to the reservoirs and microfluidic chips were placed on a rocker set at 10° and a 60 minutes interval (MIMETAS, The Netherlands). Medium was refreshed daily for 2 or 3 consecutive days for comparison between OCT and confocal microscopy, and the experiment with the treatments, respectively.

#### Treatments

After day 2 of culture, the medium in the microfluidic chips was changed to: i) control medium consisting of EGM-2 medium supplied with 2.5 μg mL^−1^ aprotinin; ii) high glucose medium with inflammatory cytokines to induce vessel regression, consisting of EGM-2 medium supplied with 2.5 μg mL^−1^ aprotinin, 30 mM d-glucose (Sigma-Aldrich, Germany), 1 ng mL^−1^ TNF-α (300-01A, PeproTech, USA) and 1 ng mL^−1^ IL-6 (A42540, Invitrogen, USA); and iii) VEGF-medium to induce vessel growth, consisting of EGM-2 medium supplied with 2.5 μg mL^−1^ aprotinin and 50 ng mL^−1^ VEGF. Each medium was refreshed daily until day 5 of culture.

#### Perfusability assay

At day 3 of culture, microfluidic chips with the control medium were measured for perfusability using 0.1 mg mL^−1^ dextran fluorescein 40 000 MW (FITC-dextran 40 kDa, D1844, Invitrogen, USA) in EGM-2 medium supplied with 2.5 μg mL^−1^ aprotinin. Microfluidic chips were imaged using the confocal laser scanning microscope before and after 7 minute incubation with FITC-dextran 40 kDa. Both reservoirs were aspirated and the FITC-dextran solution was added to one of the reservoirs to create a pressure gradient which enabled flow through the vascular network.

#### Immunostaining

Microfluidic chips were washed three times with DPBS and fixed in 4% formaldehyde solution (Sigma-Aldrich, Germany) for 1 hour at room temperature. After washing off the 4% formaldehyde solution, the vessels in the microfluidic chip were permeabilized using 2% bovine serum albumin (BSA, Sigma-Aldrich, Germany) with 0.1% Triton-X100 (Sigma-Aldrich, Germany) in PBS (with calcium and magnesium) for 1 hour at room temperature, followed by overnight at 4 °C. The vessels were then stained using primary antibodies (goat anti-VE-cadherin/CD144 (1 : 100, AF938, R&D Systems, USA) and rabbit anti-TAGLN/SM22 (1 : 200, ab14106, Abcam, UK)) in 0.2% BSA + 0.1% Triton-X100 in PBS and incubated overnight at 4 °C. The samples were then washed with 0.1% Triton-X100 and 0.1% Tween-20 in PBS four times every 1–2 hours at room temperature. The secondary antibodies (chicken anti-rabbit Alexa Fluor 488 (1 : 500, A21441, Invitrogen, USA) and donkey anti-goat Alexa Fluor 546 (1 : 500, A11056, Invitrogen, USA)) with DAPI (5 ng mL^−1^, D1306, Invitrogen™, USA) were diluted in 0.2% BSA with 0.1% Triton-X100 in PBS and incubated overnight at 4 °C. The samples were then thoroughly washed with PBS four times every 1–2 hours at room temperature and stored at 4 °C until imaging with the confocal microscope.

## Imaging

### Bright-field and confocal fluorescence imaging

Brightfield images were taken on the EVOS FL Auto 2 (Invitrogen, USA) using a 4×/0.13 Plan-Neofluar objective (Fig. 1E). Wide-field fluorescence microscopy was performed using the Zeiss Axio Observer Z1 (Carl Zeiss AG, Germany) equipped with the 10×/0.30 Plan-Neofluar objective. To excite the fluorophores, we used Zeiss Colibri LEDs (Carl Zeiss AG, Germany) 469/38 nm and 555/30 nm. A tile scan was performed on an area of 2 mm × 2 mm from the center of the microfluidic chip (Fig. 1F). For the perfusability assay, we used the Evident FV4000 laser scanning confocal microscope equipped with the 4×/0.16 U plan S-Apo objective (Olympus, Japan). Imaging was performed using the 488 nm laser line in an area of 1024 × 1024 pixels (3181.98 × 3181.98 μm) with a *z*-stack of 19 slices of each 20 μm. Images were processed in FIJI.^34^ For confocal imaging, we used the Zeiss LSM 880 Confocal Laser Scanning Microscope (Carl Zeiss AG, Germany) equipped with the 10×/0.30 Plan-Neofluar objective. Imaging was performed in AiryScanFAST mode^35^ using laser lines 488 nm and 561 nm in an area of 2568 × 2568 pixels (846.24 × 846.24 μm) with a *z*-stack of 138 slices of each 2.48 μm. Confocal images were further processed in Imaris (Oxford Instruments, USA).

#### Optical coherence tomography system

OCT images were acquired with a home-built high resolution OCT system optimized for visible light and schematized in Fig. 1D.^29,36^ Briefly, light emitted by a supercontinuum broadband source (SuperK EXTREME EXB-6, NKT Photonics, Denmark) was attenuated by three neutral density filters (ND01A, ND02A, ND03A, Thorlabs, USA). A collimator composed by three lenses (L1: LD2746-A, L2: LD2060-A, L3: LB1471-A, Thorlabs, USA) expanded the beam, while the near-infrared wavelengths (>700 nm) were filtered out by a short-pass filter (F: FESH0700, Thorlabs, USA).

An interferometer based on a 10 : 90 beam splitter (BS: BS028, Thorlabs, USA) guided 90% of the incoming light to the reference arm, where it was reflected back by a mirror (PF10-03-P01, Thorlabs, USA) to the beam splitter. A variable neutral density filter (NDF: NDC-50C-4M, Thorlabs, USA) and a dispersion compensation glass (LSM03DC-VIS, Thorlabs, USA) were placed in the reference arm, allowing control of the beam's power and dispersion. The other 10% of the incoming light was guided to the sample arm, where part of the light was backscattered by the sample of interest towards the beam splitter. Before reaching the sample, the light propagated through a neutral density filter equal to the one in the reference arm. The filter ensured optical safety for the cells by reducing the delivered power to <5 mW. A galvanometer scanner (GS: 8320 K, Cambridge Technology, USA) steered the position of the beam on the sample through the transversal *xy*-plane of a scanning lens (GS: LSM03-VIS, Thorlabs, USA). The lens had a focal length of 39 mm, a focal spot size of 12 μm, and focused the beam on the bottom of a 96-well plate holder where the chips were located.

The 96-well plate was positioned on a 3D custom-stage (Fig. 1D) composed by two *xy* translational screws and a motorized *z*-stage (T-LS13M, Zaber, USA). Light reflected by the reference mirror and backscattered by the VoCs recombined at the beam splitter and was guided to a single-mode fiber (S450XP, Thorlabs, USA). The fiber delivered the light onto a spectrometer (HoloSpec f/1.8i, Kaiser Optical Systems, USA), where a grating decomposed the light spectrum on a line camera (Sprint spL4096-140 km, Basler, Germany). The camera had an exposure time of 98 μs at a line rate of 10 kHz. The spectrometer had a spectral resolution of 0.1 nm and spanned a spectral range of 450–650 nm. The collected spectrum had a full width at half-maximum of 89 nm centered at 550 nm, implying a theoretical axial (*z*-axis) resolution for OCT imaging of ∼1.8 μm in air.

### OCT data acquisition

The microfluidic chips were transported from the culture lab to the OCT system and placed on the 3D stage. The stage was manually operated to locate the circular core of the chips near the center of the beam. The stage was slightly declined along the *z*-axis by an angle of ∼5° to prevent specular reflection when imaging from the bottom of the chips. To minimize the effect of environmental factors on the cells, chips were kept out of the incubator <30 minutes. During that time, 3D OCT scans were performed on at least *n* = 4 individual chips for the control and the two treatments. The acquisition of an OCT data set lasted approximately 3.5 minutes using a conventional raster scan. OCT cross-sectional images (B-scans, *xz* plane) spanned 2.5 × 0.9 mm, consisted of 384 × 1024 pixels (in *x* and *z* respectively), and were averaged six times for increased signal-to-noise ratio. Volumetric OCT data was obtained by displacing the beam along the *y*-axis over a range of 2.5 mm, leading to a total volume of 2.5 × 2.5 × 0.9 mm^3^ (along the *xyz*-axes, respectively) with a sampling of 384 × 384 × 1024 pixels (in the corresponding axes).

### Data processing

**Fig. 2:**
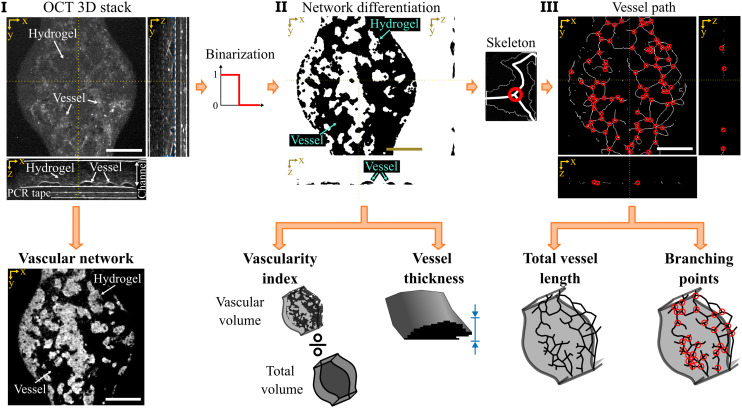
Image processing and analysis for the data retrieved from optical coherence tomography. I) Raw optical coherence tomography intensity images displaying *en-face* (*xy*-axis) and cross-sectional (*zx*- and *zy*-axis) views of the vessel-on-chip. The vascular network becomes clear with a minimum intensity projection (below). Due to the culture medium inside the vessels, the appearance of the vessels is dark while the hydrogel mixture exhibits a higher intensity signal. II) A binarization process converts the intensity images into binary images to facilitate vessel segmentation. III) A skeletonization process further reduces the binary data from II to a path-based wire image, which highlights the vessel trajectory. From II and III, we obtain the outcome measures vascularity index, vessel thickness, total vessel length, and branching points of the vascular network. Scale bar = 500 μm.

The raw OCT data underwent conventional processing to create 3D image stacks.^3,37^ Briefly, the background spectrum at the spectrometer was estimated by averaging all the raw OCT spectra and subtracted from each measurement. Then, the data set was linearized from the wavelength domain given by the spectrometer to the equivalent wavenumber domain. A Fourier transform along the wavenumber axis converted spectral domain data into spatial, depth-dependent information. From the transformed data, cross-sectional (*xz*) and *en-face* (*xy*) views were created to assess the formation of the vascular networks in the chips (as exemplified in Fig. 2-I). From the image stack, the vascular network was visualized by analyzing the higher signal intensity produced by the cells in the hydrogel suspension, compared to the lower signal intensity in the vascular network. The low signal inside the vessels arose from the lack of scattering particles in the cell medium, while the components of the hydrogel suspension scattered the incoming light.^38,39^ Two-dimensional images of the vascular network were created by projecting and normalizing the minimum signal intensity within the height of the chip (150 μm in the vascular area along the *z*-axis) containing vessels, as illustrated by the blue dashed box in the *zy* image in Fig. 2-I. OCT images of the vascular network formation were obtained for each chip over a period of 4 consecutive days.

Quantitative properties of the vascular network during formation were derived from the OCT images. Due to the low signal in the vascular network, a binarization process was applied to differentiate vessels from surrounding hydrogel (Fig. 2-II).^40–42^ The binarization consisted of a threshold at the median intensity value between the hydrogel and the vessels. As represented in Fig. 2-II, the vascular network can be isolated following this procedure and individual properties of the vessel can be analyzed. From the binarized volume, two main properties of the network were measured: (i) the vascularity index,^43^ defined as the ratio between the vascular volume to the total volume, and (ii) the vessel thickness,^44^ calculated as the vessel height along the *z*-axis.

Additional properties of the network were obtained by implementing a skeleton algorithm to the binarized network, using the function *bwskel* in MATLAB (R2024b, MathWorks, USA). The skeleton provided information of the three-dimensional vessel path by reducing the binary vascular network into a traceable path-line (Fig. 2-III). The path-line was used to obtain (i) the total vessel length defined as the sum of the total length of the vessel path-line,^45,46^ and (ii) to count the total number of branching points in the volume by evaluating the junctions where three or more paths were confluent. The latter procedure was also performed in MATLAB, using the function *branchpoints3*. Due to the non-invasive nature of OCT, the set of quantitative properties was measured every day for each chip during treatment over a period of 4 consecutive days.

### Statistical analysis

All data were plotted in boxplots and assessed using one-way analysis of variance (ANOVA) followed by a Student's *t*-test in MATLAB (R2024b). * indicates *p* < 0.05. Four individual microfluidic chips were used per condition.

## Results

### Optical coherence tomography imaging of vessel formation over time

**Fig. 3:**
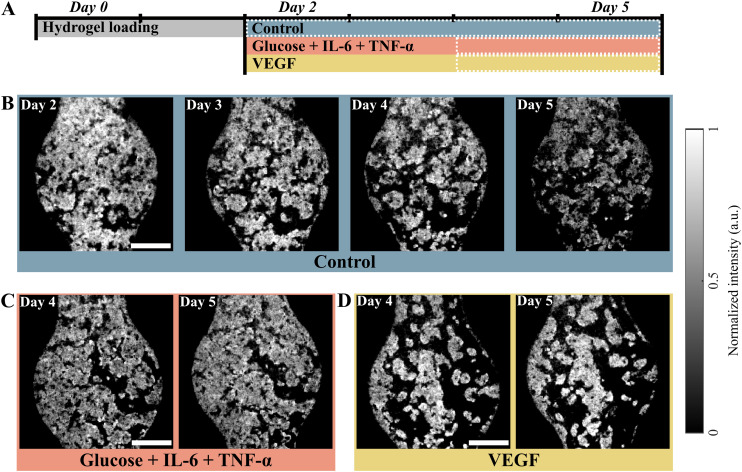
Time-resolved imaging of vascular network development in vessel-on-chip by optical coherence tomography. A) Timeline of disease modeling. Vessel-on-chips were loaded and vessels were allowed to grow for 2 days. Thereafter, the vascular network was subjected to control medium, medium with high glucose and added TNF-α and IL-6, and VEGF medium for 3 more days. Vessel-on-chips were measured every day after day 2. B) Minimum intensity projections, showing the change in the vascular network in the control condition over the course of 5 days. C) Minimum intensity projections, displaying changes in vascular network for the high glucose condition on day 4 and 5. D) Minimum intensity projections, exhibiting changes in the vascular network for the VEGF condition on day 4 and 5. For a full overview of the process, see Fig. S2 in SI. Representative images shown, scale bar = 500 μm.

To evaluate the capability of OCT to monitor the development of vessel networks over time, we performed experiments exposing the VoC to different conditions. The experiment lasted six days in total, starting with the loading and seeding of the VoCs on day 0, followed by the OCT imaging and treatment from day 2 to day 5, as outlined in Fig. 3A.

For the control, VoCs were given EGM-2 culture medium to maintain a stable environment for the vessels. This environment promoted little to no changes in vessel development during the experiment, providing a reference condition. Fig. 3B reveals that the vascular network slowly continued to grow from day 2 to day 4. The vascular network was shown to be fully perfusable at day 3 of culture (Fig. S1). On day 4, the vascular growth receded and the network appeared to be stable on day 5.

To induce vessel regression, culture medium was supplied with a high glucose concentration, IL-6 and TNF-α to emulate grade 4 serum hyperglycemia, as proposed by Maurissen *et al.*^24^ These compounds slightly hindered vascular formation on day 2 and day 3 (Fig. S2 in the SI document for the full timeline). Due to the harmful cell environment, the vascular area and the network seemed to abate on days 4 and 5 compared to the control case. This inadequate vascular network is visible in Fig. 3C, where the deficient network on day 5 displayed traces of thinning and vascular loss with respect to day 4.

To induce vessel growth, VoCs were supplied with culture medium with a high VEGF concentration to simulate CNV as found in wet-AMD.^22^ The addition of VEGF allowed for a prominent development of the network on day 2 and day 3 (see Fig. S2 in the SI). The continuous delivering of additional growth factor promoted thickening and expansion of the vessels over the duration of the experiment. Fig. 3D shows that the expansion of the vascular network continues on day 4 and day 5, possessing the larger vessels and branch connections on the latest day compared to the control case.

### Optical coherence tomography to display quantitative changes in vascular parameters

In addition to providing label-free images of the VoCs, OCT was used to quantify relevant vascular properties during formation and treatment, including vessel thickness, total vessel length, and branching points of the vascular network.

**Fig. 4:**
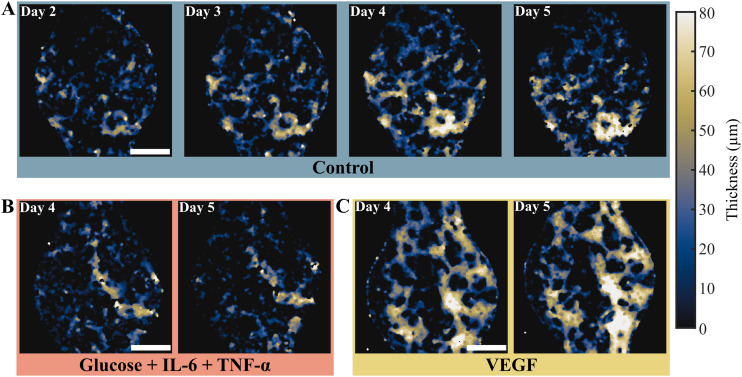
Change in vessel thickness in the vessel-on-chip over the treatment period, for the different treatments. A) Control condition on day 2 to 5, B) high glucose with added TNF-α and IL-6 condition on day 4 to 5, and C) VEGF treatment on day 4 to 5. For a full overview of the process, see Fig. S3 in SI. Representative images shown, scale bar = 500 μm.

**Fig. 5:**
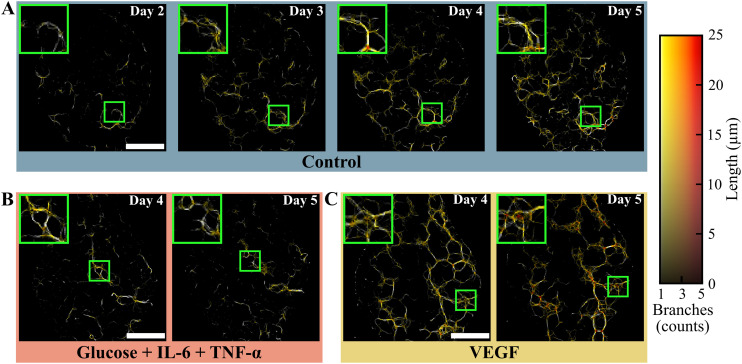
Overlay of the variation in the number of branches and vessel length under the different conditions during treatment of the vessel-on-chip for A) the control condition on day 2 to 5, B) the high glucose with added TNF-α and IL-6 condition on day 4 and 5, and C) the VEGF condition on day 4 to 5. For a full overview of the process, see Fig. S4 in SI. Representative images shown, scale bar = 500 μm.

After binarization, the OCT data was used to create quantitative maps of the local vessel thickness, as shown in Fig. 4. These maps represent the vessel thickness along the *z*-axis as a color-coded value. Overall, the thickness maps support the visual findings from the conventional OCT intensity images (Fig. 3) with additional quantitative data. These results highlight that the high glucose treatment stagnated vessel thickness, while the VEGF treatment produced a thickening of the vascular network compared to the control reference values. Furthermore, quantitative OCT data was also used to examine the path and connectivity of the vascular network during formation. These properties are displayed in Fig. 5 as a hue-luminance overlay, where the number of branches connected at every point is a variation in color (hue) and the length in depth (along the *z*-axis) the intensity (luminance). The negative impact of the high glucose treatment was observed in Fig. 5B: the vascular network shrank and its degradation led to broken connections resulting in a lower overall vascular density. In contrast, VEGF treatment exhibited much larger networks with more abundant branching points that continued to enrich even on day 5 (Fig. 5C).

### Reproducibility of the quantitative properties of the vascular network

**Fig. 6:**
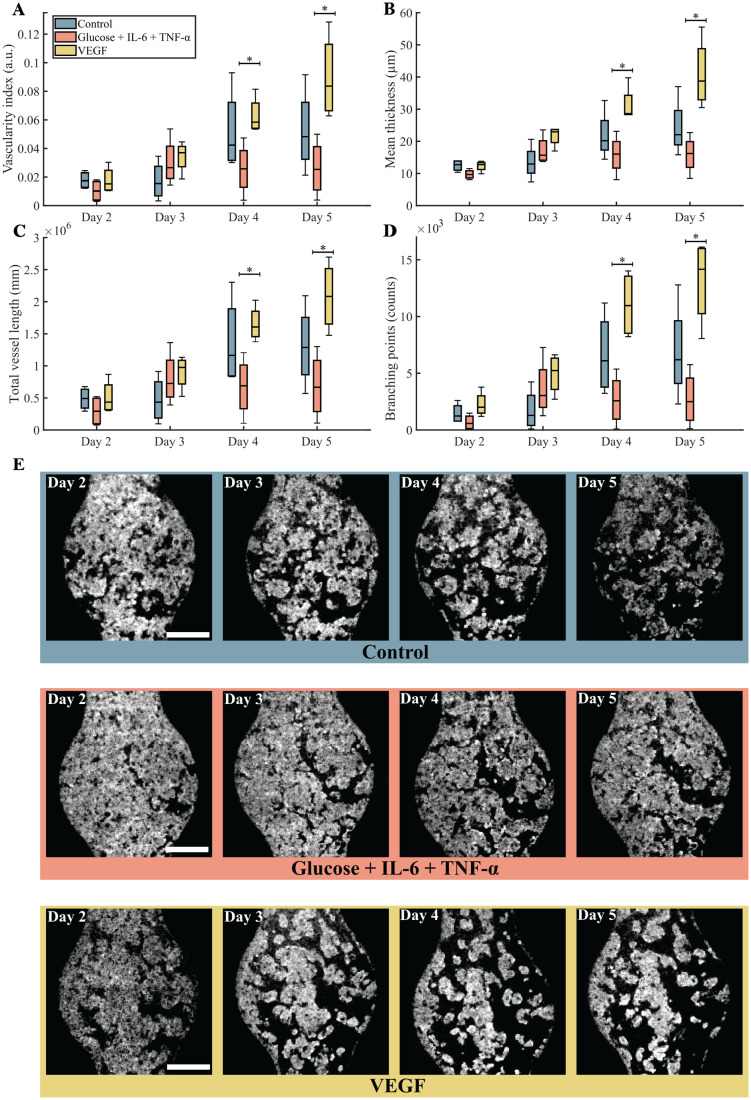
Quantitative properties of the vascular network during treatment for all conditions. A) Vascularity index (VI), B) mean thickness, C) total vessel length, and D) number of branching points. Data are presented in boxplots from four individual microfluidic chips (*n* = 4). Statistical analyses were performed using one-way ANOVA followed by a Student's *t*-test. * indicates *p* < 0.05. E) Minimum intensity projections from Fig. S2 showing the change in the vascular network in the control, high glucose with added TNF-α and IL-6, and VEGF condition over the course of 5 days. Representative images shown, scale bar = 500 μm.

To evaluate the reproducibility and significance of the VoCs, imaging and treatment, we performed a total of *n* = 4 individual experiments for each treatment condition (12 independent measurements). In each experiment, the OCT data were processed following the same methodology and the VoCs were subjected to the same experimental conditions. For each measurement, we computed the mean of the four parameters described in the Data processing section (*i.e.* i) vascularity index, ii) mean thickness, iii) total vessel length and iv) branching points). Fig. 6 presents quantitative results of each experiment during treatment for the control condition; high glucose condition, IL-6 and TNF-α mixture condition; and the VEGF condition. Quantitative measurements reveal similar growth between conditions in the first 3 days of culture. The differences between the conditions seem to appear from day 4 of culture.

### Validation of optical coherence tomography imaging with confocal microscopy

**Fig. 7:**
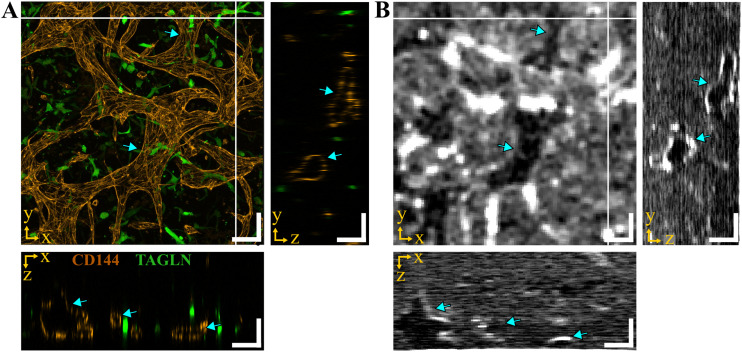
Comparison of the vascular network in the vessel-on-chip between laser scanning confocal fluorescence microscopy and optical coherence tomography. A) Maximum intensity projection of the vascular network as imaged by the laser scanning confocal fluorescence microscope. Endothelial cells are stained for CD144/VE-cadherin and vascular smooth muscle cells for TAGLN/SM22. B) Minimum intensity image from optical coherence tomography, in which vessels appear darker compared to the surrounding cells and hydrogel. The blue arrows show similar vessel structures in the projections per plane, scale bar = 100 μm.

To verify that we adequately identified vessels and the vascular network in the OCT images, we compared the OCT images with those generated by a confocal microscope (Fig. 7). In short, the VoCs were cultured for 3 days and fixed afterward. Then, OCT images were taken before continuing the immunostaining procedure. VoCs were stained for VE-cadherin (CD144) and transgelin (TAGLN) as markers for ECs and VSMCs, respectively. Then, a maximum intensity projection for the confocal microscopy and a minimum intensity projection for the OCT were created to be able to compare the results.

As shown in Fig. 7A, CD144 expression revealed the vascular network created by ECs. Proper open vessels were found, as indicated by the blue arrows in the orthogonal projections. Furthermore, VSMCs showed expression of the transgelin protein (TAGLN) and were found to be in close proximity to the ECs.

Fig. 7B shows structures of the vascular network in the minimum intensity OCT image, as indicated by the blue arrows. Thicker vessels appeared in black, while thinner vessels appeared in bright white which might be caused by the limit of the resolution of the OCT. Interestingly, similar vessel structures can be found in both *xy*-projections. In the orthogonal projections (*xz*-plane and *yz*-plane), open vessel structures can be found. Again, comparable vessel structures, as indicated by the blue arrows, were found with respect to the confocal images. Furthermore, the orthogonal projections in Fig. 7B, reveal the highly reflective layer of endothelial cells. Although the resolutions in both orthogonal projections seemed similar, the non-invasive imaging capabilities of OCT are possible at the expense of lower transversal resolution compared to microscopy imaging.

## Discussion

Visualizing and analyzing vascular networks in OoC devices generally rely on time-consuming confocal fluorescence microscopy which requires fixation and immunostaining of the sample.^22–24^ In addition to the time-consuming process, confocal fluorescence microscopy is a form of end-point analysis that limits the tracking of developments and changes of the same sample over time. To overcome this issue, we employed OCT and a microfluidic device to qualitatively and quantitatively assess the development of 3D vascular networks on a chip. Although OCT has been used for certain applications in OoC devices,^26,28–30^ it has never been used to perform real-time imaging and end-point vessel analysis. In this work, we showed qualitative tracking of vascular development, stabilization, and degradation using OCT imaging. Furthermore, we demonstrated quantitative assessment of data and retrieval of important vascular parameters, such as vessel thickness, length, and number of branching points. Aside from known quantities, we developed a method to measure the vascularity index in a microfluidic device. Despite the differences in working principle and imaging requirements for confocal microscopy (fluorescence) and OCT (light scattering), our results demonstrate that OCT can identify vessel structures similar to those of confocal microscopy at a faster image acquisition time (5 minutes of imaging time with OCT *versus* 45 minutes of confocal imaging). Another interesting aspect is the penetration depth of both imaging modalities. While the penetration depth of confocal microscopy in dense, scattering tissues, such as vessels in hydrogels, is limited,^3,47^ OCT offers a penetration depth of 1 to 3 mm, enabling visualization of future complex OoC models that include 3D vascular networks.^3^ Collectively, we demonstrated the potential of OCT imaging by combining OCT with a VoC which enabled live tissue, non-destructive, label-free imaging over multiple consecutive days.

Current analyses of *in vitro* vascular networks are usually derived from fluorescent confocal microscopic images, which typically require fixation and labeling of the vascular network. This type of analysis is performed at the end of the experiment, which means that no further analysis of that particular sample can be performed, limiting the ability to compare changes within the same sample. To enable live imaging of vascular networks, other researchers demonstrated the use of green- or red-fluorescent protein (GFP/RFP)-labeled ECs for imaging dynamics of the vascular network^48^ and demonstrated the use of optical diffraction tomography (ODT) for live and label-free imaging of VoCs.^49^ Although both techniques could visualize time-lapsed vessel dynamics at high resolution, these techniques are limited by the need for cell transfection and its limited penetration depth, respectively. To resolve issues regarding penetration depth, others have enabled fast multifocus phase imaging by combining multifocus oblique back-illumination microscopy with a *z*-splitter-based microscope.^50^ The system was used to measure blood flow and visualize blood vessels in chicken embryos in a field of view of 546 × 546 × 137 μm^3^ without labeling. More recently, Zhang, J. *et al.* introduced Gradient Retardance Optical Microscopy (GROM) for quantitative phase imaging and demonstrated label-free imaging of 300 μm-thick plant roots at high resolution which was able to unravel features at the cellular level.^51^ In our current work, we could not reveal features at the cellular level with OCT, but others have demonstrated that OCT can be employed for this purpose when incorporating contrast agents.^52^ Another advantage of OCT compared to other imaging modalities is its commercial availability and that it is widely used in the clinic, which could allow a more straightforward comparison between what is measured in the clinic and the laboratory.

The label-free imaging of our OCT set-up exposes cells to laser light, which could have biological effects. Previous studies have shown that specific wavelengths have differential effects on the viability of fibroblasts in a dose-dependent manner. More specifically, viability decreased with a power output of 23 mW and exposure times of 2 hours using lasers at 632.8 nm.^53^ Furthermore, endothelial cells in fibrin hydrogel were found to proliferate and migrate more under red and green light, but seemed to be affected in their metabolic activity and formation of reactive oxygen species under blue light with exposures once every 24 hours for 10 minutes using a dose of 40 mW per cm^2^.^54^ Since the broadband laser used in our work has a significantly lower output power of 5 mW and the exposure times are limited to 5 minutes per 24 hours, we expect that the impact of light on our culture system will be limited or negligible.

For quantifying network properties, images are often analyzed using freely available software programs, such as AutoTube and the DiameterJ plugin in ImageJ.^55,56^ Although confocal imaging can be performed in 3D using *z*-stacks, both software programs use collapsed *z*-stack images or maximum intensity projections as input and analyze from there.^33,57^ Our novel approach allowed live tissue imaging and analysis in three dimensions, allowing a more complete evaluation of vessel parameters.

In patients with diabetes mellitus, extreme plasma glucose levels (>33 mmol L^−1^) and inflammatory cytokines TNF-α and IL-6 are known as a hyperglycemic hyperosmolar state (HHS). HHS is a life-threatening emergency for patients with type 2 diabetes mellitus with high mortality rates when left untreated.^58^ The conditions used in our study simulate grade 4 serum hyperglycemia which is close to the values found in HHS and in accordance with the levels used by Maurissen *et al.*^24^ Using these conditions, we observed vessel regression in our VoC model within a few days that was detected successfully using OCT. Changes in certain vascular parameters, such as the vascular area, showed similarity to that found in other VoC models upon their stimulation with pro-inflammatory culture conditions.^23,24^ However, our model showed a faster vessel regression compared to others.^23,24^ This could be due to the relatively immature state of our vessels at the time of treatment. In other studies, extreme glucose levels were only introduced after establishing the vascular network.^23,24^ In our case, extreme glucose levels were added as our vessels were still in development. During this phase, the addition of extreme glucose concentrations probably led to an increase in oxidative stress during development, which eventually led to a faster regression of the vessels.^59,60^ In addition, we found that our iPSC-derived endothelial cells generally live shorter (up to 7 days) in fibrin hydrogel compared to well-established cell lines,^57,61^ such as human umbilical vein endothelial cells (HUVECs) or human retinal microvascular endothelial cells (HRMVECs) that were cultured up to 51 and 28 days, respectively.^24,62^ As there are known differences between iPSC-derived ECs and ECs from other sources,^63^ possible further organ-specific differentiation could improve the maturation, function, morphology and lifetime of resulting vascular networks.^52^ Another method to extend vessel lifetime could be by adding continuous flow to the model. In a similar model, it was found that continuous flow extended lifetime of vessels and induced recovery of regressed vessels.^62^ Therefore, developing organ-specific mature cells and culturing them for an extended period of time under continuous flow could help in modeling chronic disease states. Only then is it possible to model diseases such as DR that develop slowly over time in mature vessels with plasma glucose levels between 7 mmol L^−1^ and 11 mmol L^−1^ in the choroid.^64^

Regarding the VEGF treatment, we observed that increased VEGF levels in culture medium could induce neovascularization in our VoC model, analogous to how increased VEGF concentrations contribute to CNV in patients with wet-AMD.^65^ Moreover, anti-VEGF therapy, such as Avastin® (bevacizumab), is a well known treatment to reduce disease progression in wet-AMD.^66^ Although typical *in vivo* concentrations of VEGF in the serum of patients with late-stage AMD are approximately 260 pg mL^−1^, higher levels of VEGF are also found in other systemic diseases, such as diabetes mellitus.^65^ In this study, we used higher VEGF concentrations to accelerate vessel growth according to how this is typically performed in similar experimental culture systems.^22,23^

In addition to well-known vascular network measurements such as vessel length and branching, we introduced the ‘vascularity index’ as a new parameter for analyzing the networks in our VoC models. The vascularity index is a measure of the vascular density in the *xz*- and/or *yz*-plane and is defined by dividing the luminal volume by the hydrogel volume. Our definition of the vacularity index is derived from the choroidal vascularity index (CVI) which is used in clinical imaging by OCT and is defined as the fraction of luminal area with respect to the total choroidal area. Over the last decade, the CVI has been clinically identified as a potential biomarker for various retinal diseases.^10–13,67,68^ As current retina-on-chip systems capture structures of the retina and include read-outs relevant to tissue health and disease,^21^ measuring the CVI could be a valuable addition as a read-out that may enable side-by-side comparisons between patients and personalized retina-on-chip systems in the future.^69^

In our study, we demonstrated that OCT could reveal similar vessel structures as compared to confocal microscopy. However, there is a significant difference in transversal resolution (*xy*) and noise between both imaging modalities.^3,25^ The lower transversal resolution (*xy*) of OCT compared to fluorescence microscopy could be considered as a limitation of the correlation that we performed. For example, thin vessels are highly reflective in OCT (Fig. 7). This might change how the binarized image is displayed and how the thickness is measured accordingly. It should be noted that OCT transversal resolution depends on the spectral properties of the light source, as well as the imaging optics. OCT systems with superior transversal resolution have been reported and applied for OoC monitoring.^28^ Whereas the OCT system in the current study and other OoC studies are based on spectral domain OCT,^26,28–30^ various other OCT modalities are reported in literature, including time-domain, swept source and *en face* OCT.^70^ Compared to the employed spectral domain system, swept source OCT can offer advantages in imaging depth and acquisition time at the cost of lower spatial resolution with the currently available swept sources,^70^ while *en face* OCT offers superior resolution at the cost of acquisition time.^71^ Depending on the specific application in OoC imaging, the most optimal OCT modality and system can be selected based on required spatial resolution, imaging depth and acquisition time.

Previous studies have shown that flow can be measured and quantified using OCT.^28,30^ Therefore, an interesting aspect for future investigation is the addition of flow to research perfusability and permeability in 3D VoCs using OCT. Although our vascular network was shown to be perfusable in the control condition at day 3, we did not measure permeability of the network or extended these measurements among the other conditions. Despite the fact that our goal was to show structural changes under various conditions, this could be considered as a limitation of the study as flow is necessary for long-term stability of vascular networks and perfusability is an important functional metric of vascular networks.^62^ To measure perfusability and permeability with OCT, a pump (or rocker) and an incubator must be incorporated in the current OCT setup. This should subsequently allow us to evaluate perfusability and permeability using contrast agents, such as Intralipid®,^72^ and potentially correlate this with the expression of tight junction and cadherin proteins in the VoC. In addition, when flow is incorporated, more functional information can be derived from the OCT signal, such as local information on (blood) flow.^29^

All things considered, we expect that using home-built or commercially available OCT setups, OCT can become a standard readout for multilayered OoCs with or without vascular networks in situations where rough structural differences can be identified and time-lapsed or time-point measurements with a high penetration depth are required.

## Conclusion

In conclusion, we presented OCT as a novel, fast, live tissue, and label-free imaging technology to assess the development and regression of vascular networks-on-chip under different conditions. We leveraged the intrinsic characteristics of the vascular network during formation to differentiate forming vessels from the hydrogel. We observed reproducible variations in vascular growth for control, high glucose, and high VEGF conditions. In those cases, OCT images elucidated visual differences in the network formation. We used the morphological features of the chips from the OCT signal to derive quantitative properties of the vascular networks. Quantitative analysis of the OCT images further demonstrated the formation of vessels with different densities, thicknesses, branches, and lengths depending on the composition of the culture medium. We observed a consistent decrease in the vascular properties of the microfluidic chips that were given high-glucose doses compared to the control group, and an even larger decrease in comparison to the group supplied with additional VEGF. We validated those results by comparing OCT images with those produced with conventional confocal microscopy imaging. From those results, we confirmed that OCT was able to image the vascular networks without the need for immunostaining nor contrast agents, at the expense of a degraded resolution in the transversal imaging plane. All things considered, we believe that OCT could serve as a new standard read-out for microfluidic chips that include a 3D vascular network, including retina-on-chip models from which data could potentially be compared to that from the clinic.

## Author contributions

Conceptualization: DV, CCV, TG, AvdM, NB. Methodology: DV, CCV, (TG, TvD). Scientific discussions: DV, CCV, TG, AvdM, NB. Experimental procedure: DV, CCV. Investigation: DV, CCV. Funding acquisition: AvdM, NB. Supervision: AvdM, NB. Writing original draft: DV, CCV. Reviewing and editing: DV, CCV, TG, TvD, AvdM, NB.

## Conflicts of interest

The authors declare no conflict of interest.

## Supplementary Material

LC-026-D5LC00927H-s001

## Data Availability

The data sets analyzed and the algorithms generated for this manuscript are available upon prior request by contacting the corresponding authors. Supplementary information (SI) is available. See DOI: https://doi.org/10.1039/d5lc00927h.
