# Supplementary material for: Label-free assessment of a microfluidic vessel-on-chip model with visible-light optical tomography reveals structural changes in vascular networks
Source: Lab Chip. 2026 Jan 30;26(6):1901–14. doi: 10.1039/d5lc00927h (PMC12874037; doi:10.1039/d5lc00927h)
Supplement: LC-026-D5LC00927H-s001 [file LC-026-D5LC00927H-s001.pdf]

Supplementary Information (SI) for Lab on a Chip.
This journal is © The Royal Society of Chemistry 2026

Supplementary file for

Label-free assessment of a microfluidic vessel-on-chip model with visible-light optical tomography reveals structural changes in vascular networks

Devin Veerman, Carlos Cuartas-Vélez, Tarek Gensheimer, Tomas van Dorp, Andries van der Meer and Nienke Bosschaart

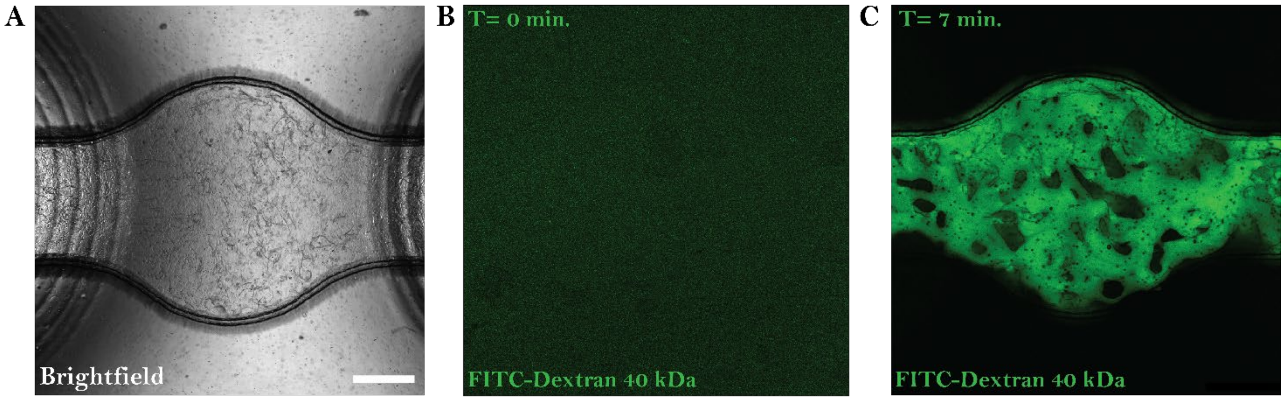

Fig. S1 Perfusability of the vascular network in the VoC at day 3 of culture. **A**) Brightfield image of the formed vessels at day 3. **B**) Maximum intensity projection of the VoC before FITC-Dextran 40 kDa was added to the left reservoir. **C**) Maximum intensity projection of the VoC after FITC-Dextran 40 kDa was added to the left reservoir and incubated for 7 minutes. Representative images shown, scale bar = 500 µm.

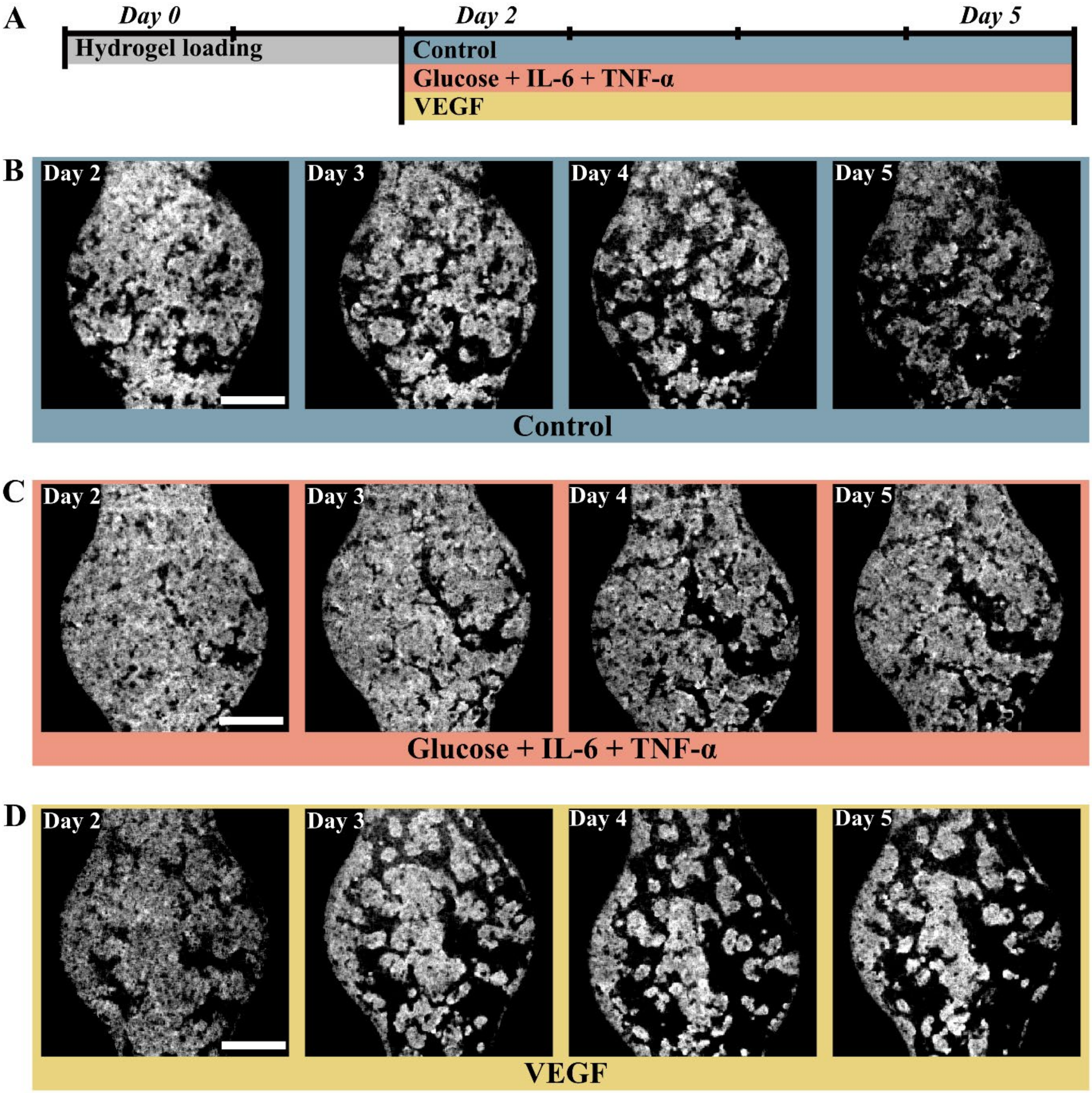

Fig. S2 Time-resolved imaging of vascular network development in vessel-on-chip by optical coherence tomography. **A**) Timeline of disease modeling. Vessel-on-chips were loaded and vessels were allowed to grow for 2 days. Thereafter, the vascular network was subjected to control medium, medium with high glucose and added TNF-α and IL-6, and VEGF medium for 3 more days. Vessel-on-chips were measured every day after day 2. **B**) Minimum intensity projections, showing the change in the vascular network in the control condition over the course of 5 days. **C**) Minimum intensity projections, displaying changes in vascular network for the high glucose with added TNF-α and IL-6 condition over the course of 5 days. **D**) Minimum intensity projections, exhibiting changes in the vascular network for the VEGF condition over the course of 5 days. Representative images shown, scale bar = 500 µm.

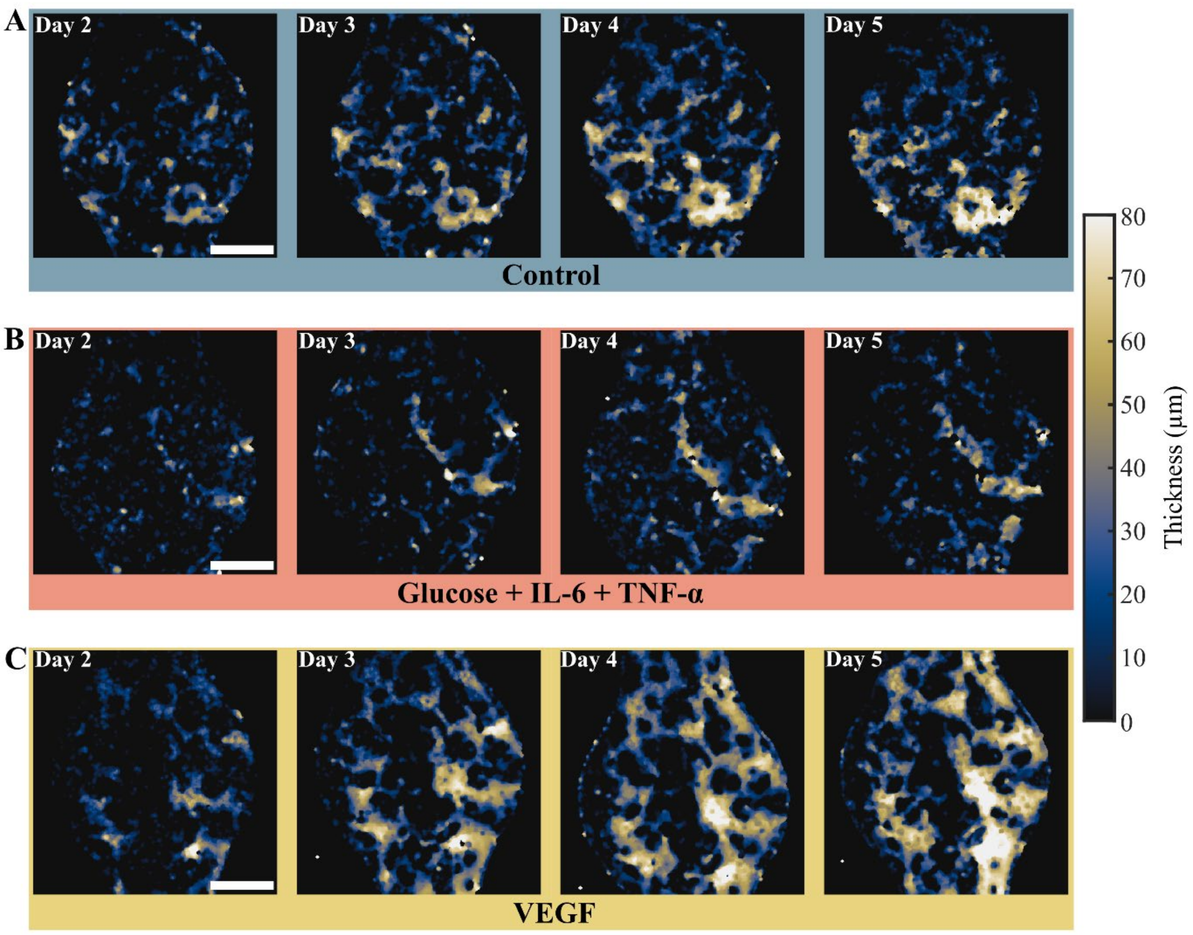

Fig. S3 Change in vessel thickness in the vessel-on-chip over the treatment period, for the different treatments. **A**) control condition on day 2 to 5, **B**) high glucose with added TNF-α and IL-6 condition over the course of 5 days, and **C**) VEGF condition over the course of 5 days. Representative images shown, scale bar = 500 µm.

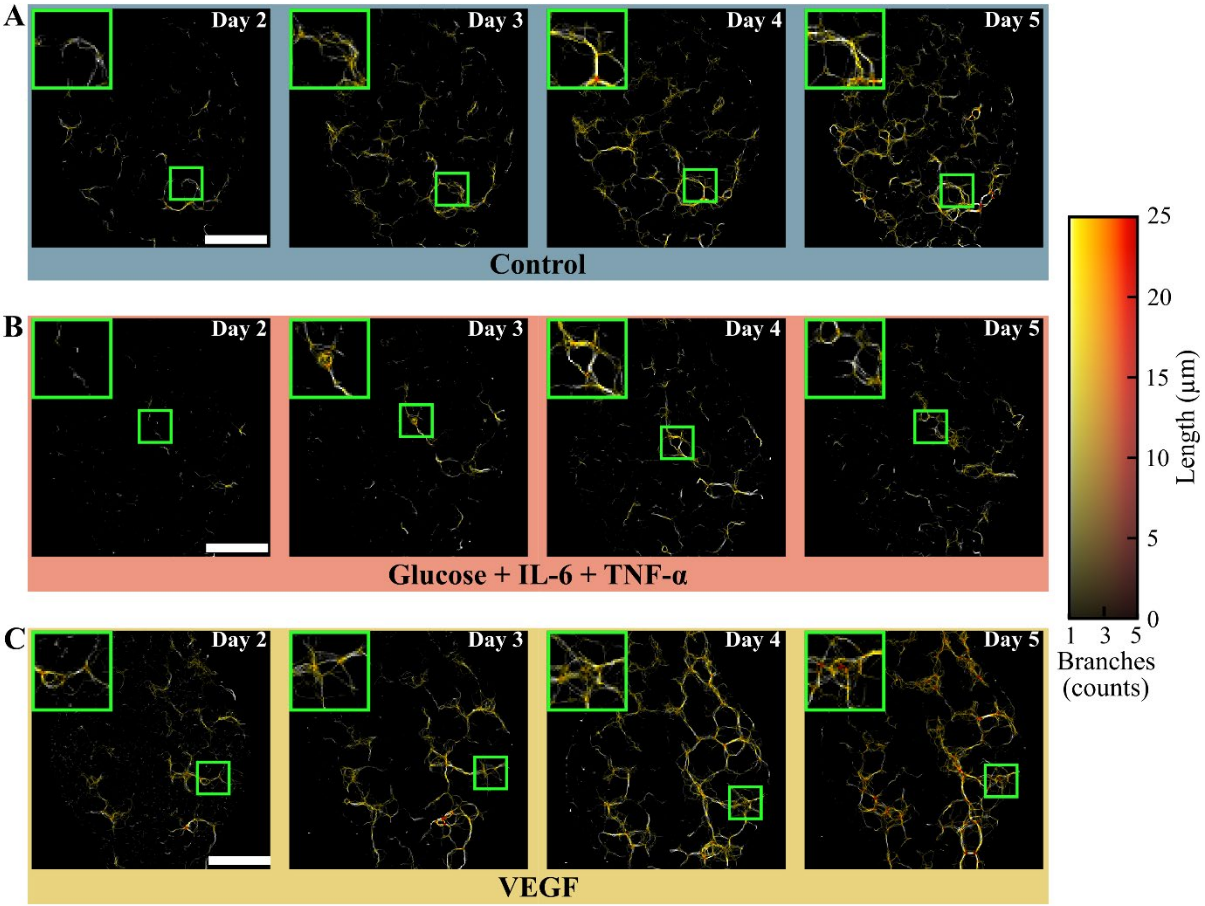

Fig. S4 Overlay of the variation in the number of branches and vessel length under the different conditions during treatment of the vessel-on-chip for **A**) the control condition on day 2 to 5, **B**) high glucose with added TNF-α and IL-6 condition over the course of 5 days, and **C**) VEGF condition over the course of 5 days. Representative images shown, scale bar = 500 μm.
